# *TIMP3* Gene Polymorphisms of -1296 T > C and -915 A > G Increase the Susceptibility to Arsenic-Induced Skin Cancer: A Cohort Study and In Silico Analysis of Mutation Impacts

**DOI:** 10.3390/ijms232314980

**Published:** 2022-11-29

**Authors:** Meei-Maan Wu, Chi-Wei Chen, Chiu-Yi Chen, Chih-Hung Lee, Mark Chou, Ling-I Hsu, Te-Chang Lee, Chien-Jen Chen

**Affiliations:** 1 Department of Public Health, School of Medicine, College of Medicine, Taipei Medical University, Taipei 11031, Taiwan; 2 Master Program in Applied Molecular Epidemiology, College of Public Health, Taipei Medical University, Taipei 11031, Taiwan; 3 School of Public Health, College of Public Health, Taipei Medical University, Taipei 11031, Taiwan; 4 Department of Life Science, College of Sciences and Engineering, National Dong Hwa University, Hualien 97430, Taiwan; 5 Department of Dermatology, Kaohsiung Chang Gung Memorial Hospital, Chang Gung University College of Medicine, Kaohsiung 83325, Taiwan; 6 Department of Research, Taiwan Blood Services Foundation, Taipei 10066, Taiwan; 7 Institute of Biomedical Sciences, Academia Sinica, Taipei 11529, Taiwan; 8 Genomics Research Center, Academia Sinica, Taipei 11529, Taiwan

**Keywords:** tissue inhibitor of metalloproteinase-3, genetic polymorphism, non-melanoma skin cancer, arsenic, molecular epidemiology, in silico analysis

## Abstract

Long-term exposure to arsenic may induce several human cancers, including non-melanoma skin cancer. The tissue inhibitor of metalloproteinase (TIMP)-3, encoded by the *TIMP3* gene, may inhibit tumor growth, invasion, and metastasis of several cancer types. In this study, we aimed to investigate effects of the *TIMP3* -1296 T > C (rs9619311) and -915 A > G (rs2234921) single-nucleotide polymorphisms (SNPs) on skin cancer risk in an arsenic-exposed population, and to evaluate the influence of allele-specific changes by an in silico analysis. In total, 1078 study participants were followed up for a median of 15 years for newly diagnosed skin cancer. New cases were identified through linkage to the National Cancer Registry of Taiwan. A Cox regression analysis was used to evaluate the effects of *TIMP3* variants. Transcription factor (TF) profiling of binding sites of allele-specific changes in SNPs was conducted using the JASPAR scan tool. We observed borderline associations between *TIMP3* genotypes and skin cancer risk. However, when combined with high arsenic exposure levels, the rs9619311 C allele, rs2234921 G allele, or C-G haplotype groups exhibited a greater risk of developing skin cancer compared to the respective common homozygous genotype group. The in silico analysis revealed several TF motifs located at or flanking the two SNP sites. We validated that the C allele of rs9619311 attenuated the binding affinity of BACH2, MEIS2, NFE2L2, and PBX2 to the *TIMP3* promoter, and that the G allele of rs2234921 reduced the affinity of E2F8 and RUNX1 to bind to the promoter. Our findings suggest significant modifications of the effect of the association between arsenic exposure and skin cancer risk by the *TIMP3* rs9619311 and rs2234921 variants. The predicted TFs and their differential binding affinities to the *TIMP3* promoter provide insights into how *TIMP3* interacts with arsenic through TFs in skin cancer formation.

## 1. Introduction

Arsenic is a ubiquitous element in the Earth’s crust. The general population is exposed to arsenic mainly through contaminated groundwater, such as that which is found in Taiwan, West Bengal, Mongolia, and some regions of the United States [1]. Long-term arsenic ingestion may induce several human cancers, including skin, lung, and urinary tract cancers [2]. Patients with chronic arsenicism present with characteristic skin manifestations of variegated hyperpigmentation, palmoplantar hyperkeratosis, and skin cancer, including Bowen’s disease, squamous cell carcinoma, and basal cell carcinoma, but not melanoma [3]. Afflicted patients also suffer from high incidences of subsequent internal cancers [4]. Although risk factors such as arsenic exposure, a poor nutritional status, and susceptible genes have been identified [5,6,7], the molecular pathogenesis of arsenic-associated cancers remains to be elucidated. Understanding the biology of arsenic-induced skin cancer may help identify people that are at risk of developing internal cancers. We previously reported that heme oxygenase (HO)-1 was involved in early-stage stress responses in an arsenic-exposed population [8], and individuals who carry a high induction genotype (short GT-repeat promoter polymorphism) have an increased risk of skin cancer and lung squamous cell carcinoma, but not lung adenocarcinoma [9]. These findings underscore the contribution of genetic susceptibility to arsenic-associated carcinogenesis. Cancer pathogenesis is a multi-step process. Therefore, we studied the biological pathways that are involved in tumor invasiveness, such as extracellular matrix (ECM) degradation, for their relationship with arsenic-associated cancers, by concentrating on skin cancer.

With ECM degradation, the matrix metalloproteinase (MMP) family has long been associated with neoplastic cell invasion and metastasis [10]. Proteolytic activities of MMPs are restrictively regulated by endogenous tissue inhibitors of metalloproteinases (TIMPs-1~4). An imbalance between the activity of MMPs and TIMPs was implicated in pathological conditions such as cardiovascular diseases, arthritis, and cancers [11]. However, TIMPs have various biological activities such as stimulating cell proliferation, pro- and anti-apoptosis, and anti-angiogenesis; these activities appear to be unrelated to their function as MMP inhibitors [12]. Among the four TIMPs, TIMP-3 is unique in that it tightly binds to the ECM, and this function is closely related to its broad metalloproteinase inhibitory activity against members of the a disintegrin and metalloproteinase (ADAM) family, which cleave cell-surface proteins and EMC molecules. TIMP-3 was shown to control tumor necrosis factor (TNF)-α-mediated inflammation and inhibit cell shedding of several molecules that are involved in the dysregulation of cell signaling pathways [13]. As TIMP-3 inhibits tumor growth, invasion, and metastasis of several cancer types, it was proposed as a human tumor suppressor [11].

TIMP-3 is the only TIMP family member that is related to an inherited genetic disease. Point mutations or splice site mutations in the C-terminal domain of the *TIMP3* gene are the cause of Sorsby’s fundus dystrophy (SFD), an autosomal dominant retinal disorder that results in macular degeneration and irreversible blindness [14]. Many recent studies have focused on other single-nucleotide polymorphism (SNP) variants that are located within the gene’s promoter or other regulatory regions in the N-terminal domain. Few of these SNPs were found to be associated with cancer development; however, some were found to be related to clinical outcomes of cancers [15,16,17,18,19,20,21]. In addition, the functional significance of most *TIMP3* variants that have been reported at present are unknown. Among up to 1500 base pairs (bp) of the *TIMP3* promoter region, three SNPs -1296 T > C (rs9619311), -915 A > G (rs2234921), and -899 T > C (rs2234920) were identified. Through a computer analysis, the region revealed a variety of consensus binding sites for transcription factors (TFs). Yet the identified polymorphisms did not alter any of the predicted binding sites for TFs, such as activator protein (AP)-1 or nuclear factor (NF)-κB [22]. The functional significance of the SNPs remains unclear, and the differential affinities resulting from base substitutions need to be evaluated.

The effects of *TIMP3* genetic variants on cancer risks were reported in the literature; however, those studies were based on cross-sectional or case-control study designs, in which differential survival probabilities may have occurred between groups of various genotypes. In addition, few studies have considered the effects of environmental factors. Herein, we conducted a cohort follow-up study to investigate the effects of *TIMP3* promoter polymorphisms on skin cancer risks in 1078 individuals from an arsenic-exposed population. Furthermore, to evaluate the impacts of the SNP variants, TF profiling for the binding sites of allele-specific changes was conducted by an in silico analysis.

## 2. Results

### 2.1. Baseline Characteristics by TIMP3 Promoter Genotypes

The three studied SNPs of -1296 T > C (rs9619311), -915 A > G (rs2234921), and -899 T > C (rs2234920) present similar allelic frequencies to those of East Asian populations (https://www.ncbi.nlm.nih.gov/snp). The rs2234920 gene locus was not included in the subsequent analysis because of a low minor allelic frequency (Table S1). The other two SNPs had a high degree of linkage disequilibrium (LD), which formed two main components of a haplotype block, T-A and C-G (Figure S1).

The distributions of the *TIMP3* genotype by SNPs are presented in Table 1. In all three genotypes, each SNP in these study participants complied with the Hardy–Weinberg equilibrium (HWE, *p* > 0.05). As shown in the table, the distribution of baseline characteristics, including age, gender, education level, cigarette smoking, alcohol consumption, and arsenic exposure, were similar among the three genotypes for each respective SNP (*p* > 0.05 for all).

### 2.2. Factors Associated with the Skin Cancer Incidence

In total, 50 patients were newly diagnosed with skin cancer during a median 15-year follow-up. As shown in Table 2, an older age, male gender, and higher levels of arsenic exposure were significantly associated with an increased risk of skin cancer in the study participants. On the other hand, a higher education level and habitual smoking were associated with a decreased risk of skin cancer. Table 3 shows the relationship between the *TIMP3* promoter genotype and the risk of skin cancer. Although not statistically significant, there was a trend toward a positive association between the skin cancer risk with carriers of the SNPs of the rs9619311 C allele and rs2234921 G allele. In the haplotype analysis, no single diplotype was significantly associated with the risk of skin cancer (Table S2).

### 2.3. Synergetic Effect of the TIMP3 Genotype and Arsenic Exposure

Although the *TIMP3* genotype was a weak independent predictor of skin cancer risk, we examined whether the genotype and arsenic exposure had an interactive effect on the skin cancer risk. As shown in Table 4, when combined with high levels of arsenic exposure, the rs9619311 C allele (HR = 3.29, 95% CI = 1.07~10.12, dominant model), rs2234921 G allele (HR = 3.81, 95% CI = 1.30~11.23, dominant model; HR = 82.52, 95% CI = 8.60~791.61, recessive model), and C-G haplotype (HR = 3.31, 95% CI = 1.08~10.18, dominant model) groups had a greater risk of developing skin cancer compared to the respective common homozygous genotype groups.

### 2.4. TIMP3 Genotype and Incidence of Skin Lesions

We further examined the relationship between *TIMP3* promoter genotypes and the incidence of skin lesions in the study participants. There was an increased risk of hyperpigmentation (OR = 6.72, 95% CI = 1.59~28.42) in the rs2234921 G/G genotype group, as shown in Table 5. The G allele of rs2234921 of the *TIMP3* gene was only marginally associated with a hyperkeratosis risk (*p* = 0.06). We also found a significant association between arsenic exposure and the risk of skin lesions in the rs2234921 G allele group (HR = 2.96, 95% CI = 1.25~6.96, dominant model; HR = 19.82, 95% CI = 4.34~90.57, recessive model), who had a greater risk of developing skin lesions compared to the common homozygous genotype group (Table 6). These results also support a synergetic effect of the *TIMP3* genotype and arsenic exposure on the skin lesion risk.

### 2.5. In Silico Analysis of TF-Binding Sites (TFBSs) and the Impacts of Mutations

**Figure 1:**
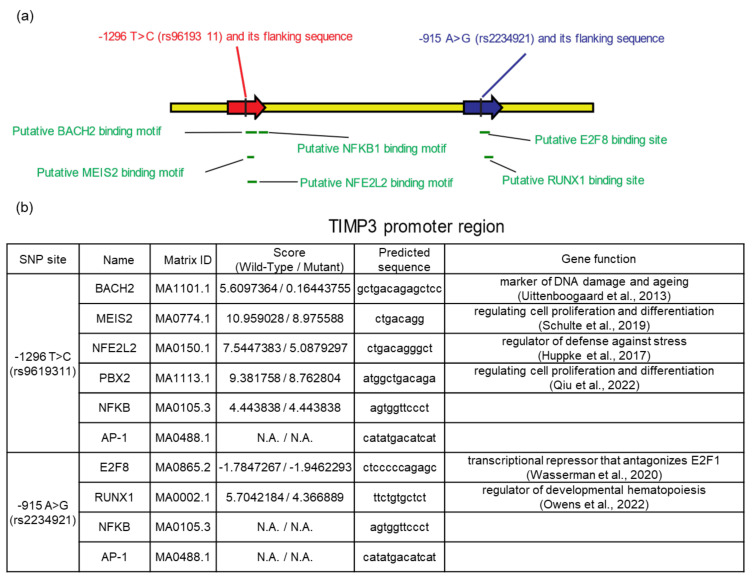
(**a**) rs9619311 and rs2234921 are, respectively, located within the predicted sequence of four and two transcription factor (TF) motifs. (**b**) TF-binding site scores were obtained using the JASPAR scan tool for the wild-type and mutant forms of each studied single-nucleotide polymorphism (SNP). For references of gene function: BACH2 [25], MEIS2 [26], NFE2L2 [27], PBX2 [28], E2F8 [29], RUNX1 [30]. N.A., not available.

To discover potential TF motifs located at or flanking the rs9619311 and rs2234921 SNPs, we launched a detailed survey using a eukaryotic promoter database [23] and the JASPAR database [24]. Among 568 TF motifs, rs9619311 and rs2234921 were found to respectively be located within the predicted sequence of 15 and 3 transcription motifs (Figure S2). To further analyze whether the T to C conversion of rs9619311 affected the binding affinities of each motif, we used the JASPAR scan tool to obtain TFBS scores and compared the scores between the wild-type (T) and mutant (C) rs9619311. Accordingly, mutant rs9619311 was found to have attenuated binding to the BACH2, MEIS2, NFE2L2, and PBX2 TFs as shown in Figure 1. Mutant rs2234921 (G allele) was also found to have a reduced binding affinity of E2F8 and RUNX1 to their motifs, as shown. Interestingly, we did not find differences in the binding affinities of NF-κB by rs9619311, although a putative NF-κB motif flanking rs9619311 was identified. In addition, we found no AP-1 motif in rs9619311 or rs2234921.

## 3. Discussion

In this study, we examined contributions of the *TIMP3* -1296 T > C (rs9619311) and -915 A > G (rs2234921) SNPs to arsenic-induced skin cancer/lesion risk. In the recessive model, we observed marginal and significant associations of the G allele of the rs2234921 SNP with the risk of arsenic-induced skin cancer and skin lesions, respectively. The rs9619311 SNP alone or in the haplotype block showed no association with the skin cancer or skin lesion risk. Although there were few associations of the studied SNPs with the skin cancer/lesion risk, we found a significant modification effect of variant alleles on the association between arsenic exposure and skin cancer/lesion risk in either the dominant or recessive model.

Previous studies on the associations between the *TIMP3* -1296 T > C and the risk of various cancers produced inconsistent results. A case-control study reported that variant genotypes (T/C or C/C) were positively associated with breast cancer risk [17]. In contrast, other studies reported associations of variant genotypes with a decreased risk of cancer, such as hepatocellular carcinoma in females and colorectal cancer [15]. However, the most recent case-control studies or patient survival studies have so far found no associations between variant genotypes and the risk of cancers, including breast [18], bladder [21], gastroesophageal junction [16], and oral cavity cancers [19]. Our study did not find a significant association between the *TIMP3* -1296 T > C SNP and skin cancer or skin lesion risks, although a borderline association of the -915 A > G SNP, an SNP in LD with the -1296 T > C site, was observed. To our knowledge, no observational studies have investigated the associations of the -915 A > G SNP with various cancers. Discrepancies in the literature may be attributed to different types of cancers, different stages of the same cancer, or other yet unidentified factors.

In the present study, we found a significant effect modification of the studied SNPs on the association between arsenic exposure and skin cancer/lesion risk. Subjects who carried a variant genotype (T/C or C/C of rs9619311 or A/G or G/G of rs2234921) were at an increased risk of skin cancer/lesions that were associated with arsenic exposure compared to those who carried the respective common homozygous genotypes (T/T of rs9619311 and A/A of rs2234921). To our knowledge, this study is the first report of an interaction between *TIMP3* genetic polymorphisms and an environmental contaminant such as arsenic. These results suggest that genetic variants of the *TIMP3* promoter region could play a role in arsenic-induced skin carcinogenesis.

Arsenic is a strong oxidant that rapidly induces several stress proteins in cultured cells [31]. During arsenic metabolism in cells, reactive oxygen species are generated [32]. Although the exact molecular mechanisms of arsenic carcinogenesis are not well understood, it is generally accepted that free radicals that are generated by arsenic may induce intracellular signal transduction and activate redox-sensitive transcription factors such as AP-1, NF-κB, and nuclear factor erythroid 2-related factor 2 (NRF2), which in turn change expressions of genes that are involved in cell growth, proliferation, and malignant transformation [33,34]. However, in this study, the in silico analysis revealed that the two studied SNPs did not predict the binding sites for either AP-1 or NF-κB. This finding is consistent with a previous report by Beranek et al. [22]. Interestingly, we found several TF motifs located at or flanking the two SNP sites of *TIMP3* that had not previously been reported. We further validated that the C allele of rs9619311 attenuated the binding affinity of BACH2, MEIS2, NFE2L2, and PBX2 to the *TIMP3* promoter. We also validated that the G allele of rs2234921 reduced the affinity of E2F8 and RUNX1 to bind to their site on the promoter. BACH2 was identified as a competitor for the Maf protein in the NRF2 antioxidant defense pathway [35] and was recently shown to be highly sensitive to DNA damage and aging [25]. MEIS2 is a homeobox protein usually in complex with PBX and HOX to promote downstream gene transcription that is involved in early development and cell differentiation [26]. Others also showed a correlation between MEIS2 and the progression of various cancers [36,37,38,39]. *NFE2L2* encodes NRF2, which is the master regulator of cell defense against stress [27], suggesting that *TIMP3* may play a role in downstream gene responses to cellular redox imbalances. PBX2 is a member of the PBX family that was initially identified as an essential Hox cofactor that plays crucial roles in early development [28]. Recent studies suggested that PBX2 dysregulation is closely associated with cancer progression [40,41]. E2F8 is a transcription repressor that antagonizes E2F1 in regulating the cell cycle, apoptosis, and tumor promotion [29]. RUNX1 was initially described as a critical regulator of developmental hematopoiesis [30]. Recently, RUNX1 was also associated with solid tumor development in the skin, lungs, intestines, and breast [42]. Taken together, the reduced affinity of the above TFs to *TIMP3* promoter variants may result in decreased *TIMP3* gene expression and activity. Although the predicted TFs and their differential binding affinities need to be confirmed by experimental approaches, our results agree with the reported functional effects of TIMP-3 on essential cellular processes, including proliferation, differentiation, and apoptosis in the regulation of cancer genesis.

The strength of this study is the prospective study design with newly diagnosed cancer/lesion cases. Unlike the case-control study design of most previous *TIMP3* SNP studies, the possibility of reverse causation could thus be minimized. In addition, this study used a national registry database to determine the incident cancer cases, which contains accurate and complete datasets that are periodically updated. Several limitations of this study should be noted. First, because of the small sample size, we could not evaluate the cancer risk by cancer subtype, such as Bowen’s disease vs. invasive skin cancer. Whether our findings differ among subtypes is not known. The small number of incident cases is also of concern; hence this study’s findings may have been due to chance. In addition, most of our results showed a modest to large effect size of the association but did not reach statistical significance. One explanation may be due to the lower minor allele frequency (MAF) observed in the two SNPs examined in our study participants. However, the MAF occurred at a similar frequency to that reported in previous studies involving Taiwanese [19,20] and as reported in the dbSNP database that includes East Asians (https://www.ncbi.nlm.nih.gov/snp). The allelic and genotypic frequencies of our study participants should have been well represented.

## 4. Materials and Methods

### 4.1. Study Participants and Questionnaire Data

The recruitment of the study participants and the collection of questionnaire data and blood samples were previously described [5,9,43]. In brief, study participants were recruited from two arseniasis endemic areas in Taiwan: a Blackfoot disease-endemic area in the southwestern region (LMN subcohort) and the Lanyang Basin in the northeastern coastal region (Lanyang subcohort). Residents of these two areas were exposed to arsenic-tainted groundwater in the 1910s~1970s and 1940s~1990s, respectively. Arsenic exposure is associated with increased risks of non-melanoma skin cancer [44,45], lung cancer [43], and urinary tract cancer [46]. Epidemiologic follow-up studies were launched in 1989 and 1998 to identify susceptibility factors for arsenic-associated diseases. Since then, periodic physical examinations to identify skin cancer and skin lesions have been carried out in the endemic areas [4,5,47].

This study consisted of 1078 study participants from the two study subcohorts who had baseline questionnaire data and successful genotyping results. These study participants were reported to have increased mortality from cardiovascular diseases and an increased incidence of a variety of cancers associated with arsenic [9,48]. A prior study identified demographic and lifestyle risk factors for cancers in the endemic areas, such as age, gender, education level, cigarette smoking, and alcohol consumption which were considered to be potential confounding factors in the present study for the association analysis [9]. Individuals were considered regular users if they smoked cigarettes or consumed alcohol at least 3 days a week for at least 6 months. To represent the overall exposure to arsenic from artesian well water for each study subject, cumulative arsenic exposure was applied as an index of arsenic exposure, calculated as previously described [4]. Informed consent was obtained for participation at the time of enrollment [9,49]. This study was performed in accordance with the Declaration of Helsinki and was approved by the institutional review boards of Taipei Medical University (N201807031, 2018 July 24) and the Genomics Research Center, Academia Sinica (AS-IRB01-09008, 6 April 2009 and AS-IRB01-11070, 3 March 2012).

### 4.2. Identification of Skin Cancer Cases

We identified newly diagnosed skin cancer patients through a data linkage with profiles of the National Cancer Registry of Taiwan and Death Certification System of Taiwan as previously described [9]. The nationwide Cancer Registry was implemented in 1979. Both invasive cancers and carcinoma in situ of the skin (Bowen’s disease) were included in the present study. In Taiwan, it is mandatory to report Bowen’s disease and cervical carcinoma in situ to the Cancer Registry. The percentage of pathological confirmation of skin cancer cases was 99.7% [50]. The Death Certification System has been computerized since 1972, and the system maintains updated information on the vital status of Taiwanese citizens. The total number of person-years (P-Y) in the follow-up period for each participant was calculated from the interview date to the date of cancer diagnosis, death, or the end of follow-up (31 December 2014), whichever occurred earliest.

### 4.3. Identification of Skin Lesion Cases

Skin lesions, including hyperpigmentation and hyperkeratosis, were clinically diagnosed during community health examinations held from 1989 to 2009 for the LMN subcohort. The total number of person-years for each participant was calculated from the date of the skin examination with no manifestation of skin lesions to the date of lesions being newly diagnosed, death, or the end of follow-up (30 April 2009), whichever occurred earliest. The vital status was determined by linking it to the Death Certification System as described above.

### 4.4. TIMP3 Promoter Genotyping

DNA were extracted from the buffy coat according to the manufacturer’s instructions (Gentra System, Minneapolis, MN, USA). The primer sequences that were used for fragment amplification were 5′-GAAAGGGGTGACGAGTTCCTG-3′ (forward) and 5′-CCTTGACTGTGCTTGGTGGA-3′ (reverse). The polymerase chain reaction (PCR) analysis was carried out in a final volume of 25 μL containing 12.5 μL of 2× Tag PCR mix (TIANGEN Biotech, Beijing, China), 1 μL of 10 μM of each primer, 0.5 μL of 10 mM dNTP, and 1 μL of a 5-ng DNA sample. The thermal cycling conditions were as follows: pre-denaturation at 94 °C for 5 min, 33 cycles of denaturation at 94 °C for 30 s, annealing at 65 °C for 30 s, and extension at 72 °C for 30 s. Finally, the process was extended at 72 °C for 5 min. All the PCR products (903 bp) were sent to the Institute of Biomedical Sciences, Academia Sinica (Taipei, Taiwan), for a sequencing analysis (Figure S3). A total of five percent of the analyzed samples were randomly selected for a reproducibility analysis, and the repeated results were 100% consistent.

### 4.5. In Silico Analysis of TF-Binding Profiles

The EPD server (https://epd.epfl.ch//index.php, accessed on 5 October 2022) is an in silico tool for analyzing promoter-binding regions [23]. The Search Motif Tool was applied to analyze TF motifs located in *TIMP3* based on bioinformatics from JASPAR (https://jaspar.genereg.net, accessed on 5 October 2022). JASPAR is an online server for profiling TF binding, position frequency matrices (PFMs), and TF flexible models (TFFMs). The score of each binding site was calculated as log(P(Site|Matrix)/P(Site|Background)) [24].

### 4.6. Statistical Analysis

For the genetic analysis, we first examined the Hardy–Weinberg equilibrium (HWE) in the study participants using the χ^2^ goodness-of-fit test. We then used Haploview v.4.2 (Broad Institute, Cambridge, MA, USA), to measure linkage disequilibrium (LD) between the studied SNPs and to build a haplotypic block. To compare the frequency distributions of baseline characteristics among the genotype groups, we used the χ^2^ test or Fisher’s exact test. To analyze the associations of the studied SNPs with skin cancer/lesion risks, we used Cox proportional hazard models while adjusting for other covariates. All variables were categorized except for age. Arsenic exposure was categorized into three groups ≤ 7.7, 7.7~17.5, and > 17.5 ppm-years with reference to previous reports [4,51]. Trend tests were estimated by treating the three exposure groups as a continuous variable, and integer scores were coded in the regression analysis. To examine the association of *TIMP3* genotypes with skin cancer/lesion risk, we calculated hazard ratios (HRs) and 95% confidence intervals (CIs) derived from Cox regression analyses based on three genetic models: additive, dominant, and recessive.

To assess whether there was an interaction between the *TIMP3* promoter genotype and arsenic exposure on skin cancer/lesion risk, we conducted a combination analysis of the two variables on the cancer/lesion risk. We examined whether the group with the risk genotype and high arsenic exposure (>17.5 ppm-years) exhibited a higher risk than the group with the risk genotype alone or high arsenic exposure alone. Additive interactions and multiplicative interactions were respectively evaluated using a trend test and a likelihood ratio test. All statistical analyses were performed using SAS 9.4 (SAS Institute, Cary, NC, USA), and two-tailed *p* < 0.05 was considered significant.

## 5. Conclusions

Based on this cohort study, our data suggest that *TIMP3* -1296 T > C and -915 A > G promoter SNPs are not likely significant predictors of skin cancer/lesion risk among Taiwanese participants. However, we found a significant effect modification of the association between arsenic exposure and skin cancer/lesion risk by variant alleles of the studied SNPs. These findings have implications for risk stratification in arsenic risk assessments. Our results of the in silico analysis provide insights into the roles of several transcription factors that bind to the two *TIMP3* promoter SNP sites. These findings may facilitate further investigation of how *TIMP3* interacts with arsenic through transcription factors and its role in cancer formation.

## Figures and Tables

**Table 1:** Baseline characteristics of study participants across *TIMP3* rs9619311 genotypes and rs2234921 genotypes.

	rs9619311 (*N* = 1078)				rs2234921 (*N* = 1072)			
Variables *	T/T (*n* = 911) ^†^	T/C (*n* = 156)	C/C (*n* = 11)	*p*	A/A (*n* = 909)	A/G (*n* = 157)	G/G (*n* = 6)	*p*
Age at enrollment, years				0.130				0.104
30~50	290 (31.8)	35 (22.4)	2 (18.2)		292 (32.1)	35 (22.3)	1 (16.7)	
50~60	301 (33.0)	55 (35.3)	4 (36.4)		299 (32.9)	58 (36.9)	2 (33.3)	
≥60	320 (35.1)	66 (42.3)	5 (45.5)		318 (35.0)	64 (40.8)	3 (50.0)	
Gender				0.356				0.670
Female	506 (55.5)	82 (52.6)	4 (36.4)		504 (55.5)	82 (52.2)	4 (66.7)	
Male	405 (44.5)	74 (47.4)	7 (63.6)		405 (44.6)	75 (47.8)	2 (33.3)	
Education level				0.427				0.117
No schooling	337 (37.0)	53 (34.0)	6 (54.6)		337 (37.1)	50 (31.9)	4 (66.7)	
Elementary	438 (48.1)	85 (54.5)	4 (36.4)		435 (47.9)	90 (57.3)	2 (33.3)	
Junior high or above	136 (14.9)	18 (11.5)	1 (9.1)		137 (15.1)	17 (10.8)	0 (00.0)	
Cigarette smoking				0.290				0.584
No	657 (72.1)	118 (75.6)	10 (90.9)		657 (72.3)	119 (75.8)	4 (66.7)	
Yes	254 (27.9)	38 (24.4)	1 (9.1)		252 (27.7)	38 (24.2)	2 (33.3)	
Alcohol consumption				0.645				0.553
No	773 (84.9)	137 (87.8)	10 (90.9)		771 (84.8)	137 (87.3)	6 (100.0)	
Yes	138 (15.2)	19 (12.2)	1 (9.1)		138 (15.2)	20 (12.7)	0 (0.00)	
Body-mass index, kg/m^2^				0.561				0.069
≤23	319 (35.4)	63 (40.9)	5 (45.5)		318 (35.4)	66 (42.6)	2 (33.3)	
23~27	388 (43.1)	64 (41.6)	5 (45.5)		387 (43.1)	66 (42.6)	1 (16.7)	
≥27	194 (21.5)	27 (17.5)	1 (9.1)		194 (21.6)	23 (14.8)	3 (50.0)	
Arsenic exposure, ppm-years				0.285				0.888
≤7.7	381 (48.6)	74 (56.9)	4 (44.4)		381 (48.7)	67 (51.9)	4 (66.7)	
7.7~17.5	201 (25.6)	29 (22.3)	4 (44.4)		200 (25.6)	33 (25.6)	1 (16.7)	
>17.5	202 (25.8)	27 (20.8)	1 (11.1)		201 (25.7)	29 (22.5)	1 (16.7)	

* All variables are categorized and presented as counts (percentage). ^†^ Differences between total counts and the total number (*n*) by genotype are due to missing data.

**Table 2:** Hazard ratios (HRs) of skin cancer in relation to baseline characteristics among the study participants.

			Age- and Gender-Adjusted		Multivariate-Adjusted *	
Characteristic	P-Y	SC Cases	HR (95% CI)	*p*	HR (95% CI)	*p*
Age	20,551	50	1.05 (1.02~1.08)	<0.001	1.05 (1.02~1.08)	0.004
Gender						
Female	11,782	22			1.00	
Male	8769	28	1.67 (0.95~2.92)	0.073	4.09 (2.09~8.03)	<0.001
Education level						
No schooling	7040	25			1.00	
Elementary or above	13,495	25	0.51 (0.28~0.95)	0.033	0.49 (0.26~0.92)	0.025
Smoking						
No	15,598	45			1.00	
Yes	4952	5	0.13 (0.05~0.34)	<0.001	0.18 (0.07~0.48)	<0.001
Drinking						
No	17,816	46			1.00	
Yes	2735	4	0.34 (0.12~0.98)	0.046	0.51 (0.17~1.49)	0.216
Body-mass index, kg/m^2^						
<27	16,179	39				
≥27	4179	11	1.12 (0.57~2.20)	0.736	(N.A.)	
Arsenic exposure, ppm-years						
≤7.7	8077	9	1.00		1.00	
7.7~17.5	4897	14	2.64 (1.13~6.17)	0.026	2.12 (0.90~5.01)	0.088
>17.5	4166	23	4.18 (1.92~9.10)	<0.001	3.17 (1.44~6.99)	0.004
Trend test			1.98 (1.38~2.84)	<0.001	1.74 (1.20~2.52)	0.004

P-Y, person-years; SC, skin cancer; CI, confidence interval; N.A., not applied. * Adjusted for the other variables as listed in the table except for the body-mass index variable.

**Table 3:** Hazard ratios (HRs) of skin cancer in relation to the *TIMP3* rs9619311 and rs2234921 genotypes.

TIMP3 Genotype	P-Y	SC Cases	aHR (95% CI) *	*p*
*rs9619311(T > C)*				
Additive model				
T/T	13,695	34	1.00	0.606
T/C	2064	7	1.25 (0.54~2.87)	
C/C	114	0	(N.A.)	
Dominant model				
T/T	13,695	34	1.00	
T/C + C/C	2178	7	1.16 (0.51~2.68)	0.722
Recessive model				
T/T + T/C	15,759	41	1.00	
C/C	114	0	(N.A.)	
*rs2234921(A > G)*				
Additive model				
A/A	13,665	33	1.00	
A/G	2050	7	1.29 (0.56~2.96)	0.551
G/G	84	1	6.71 (0.85~53.20)	0.071
Dominant model				
A/A	13,665	33	1.00	
A/G + G/G	2134	8	1.43 (0.65~3.16)	0.372
Recessive model				
A/A + A/G	15,715	40	1.00	
G/G	84	1	6.42 (0.81~50.71)	0.078

P-Y, person-years; SC, skin cancer; aHR, adjusted hazard ratio; CI, confidence interval; N.A., not available. * Adjusted for age, gender, education level, cigarette smoking, and arsenic exposure.

**Table 4:** Combined effects of *TIMP3* genotypes and arsenic exposure on the risk of skin cancer.

TIMP3 Genotype	Arsenic Exposure *	Cases/P-Y	aHR (95% CI) ^†^	*p*
*rs9619311*				
Dominant				
T/T	Low	7/6420	1.00	
	High	27/7274	2.35 (1.00~5.53)	0.051
T/C + C/C	Low	1/1205	0.52 (0.06~4.27)	0.542
	High	6/973	3.29 (1.07~10.12)	0.038
		Trend test	1.82 (1.06~3.13)	0.030
		Multiplicative test	1.41 (0.57~3.46)	0.455
*rs2234921*				
Dominant				
A/A	Low	7/6412	1.00	
	High	26/7253	2.26 (0.96~5.34)	0.063
A/G + G/G	Low	1/1124	0.64 (0.08~5.28)	0.682
	High	7/1010	3.81 (1.30~11.23)	0.015
		Trend test	1.95 (1.14~3.32)	0.015
		Multiplicative test	1.69 (0.72~3.95)	0.226
Recessive				
A/A + A/G	Low	8/7481	1.00	
	High	32/8234	2.52 (1.13~5.62)	0.024
G/G	Low	0/54	N.A.	
	High	1/30	82.52 (8.60~791.61)	<0.001
		Trend test	2.98 (1.31~6.77)	0.009
		Multiplicative test	32.50 (3.68~286.77)	0.002
*Haplotype block*				
Dominant				
T-A/T-A	Low	7/6380	1.00	
	High	26/7228	2.16 (0.92~5.12)	0.079
T-A/C-G + C-G/C-G	Low	1/1030	0.71 (0.09~5.82)	0.750
	High	6/929	3.31 (1.08~10.18)	0.037
		Trend test	1.82 (1.05~3.14)	0.032
		Multiplicative test	1.53 (0.62~3.78)	0.354

P-Y, person-years; aHR, adjusted hazard ratio; CI, confidence interval; N.A., not available. * Low arsenic exposure indicates ≤7.7 ppm-years; ^†^ Adjusted for age, gender, education level, and cigarette smoking.

**Table 5:** Hazard ratios (HRs) of skin lesions (hyperkeratosis and hyperpigmentation) in relation to the *TIMP3* rs9619311 and rs2234921 genotypes.

TIMP3 Genotype	P-Y	Hyperkeratosis	aHR (95% CI) *	*p*	P-Y	Hyperpigmentation	aHR (95% CI)	*p*
*rs9619311(T > C)*								
Additive model								
T/T	3707	52	1.00		3246	94	1.00	
T/C	391	10	1.93 (0.96~3.89)	0.064	381	11	0.90 (0.48~1.70)	0.752
C/C	27	0	(N.A.)		14	2	3.51 (0.82~14.96)	0.090
Dominant model								
T/T	3707	52	1.00		3246	94	1.00	
T/C + C/C	419	10	1.75 (0.87~3.52)	0.116	395	13	1.02 (0.56~1.83)	0.962
Recessive model								
T/T + T/C	4098	62	1.00		3627	105	1.00	
C/C	27	0	(N.A.)		14	2	3.56 (0.84~15.15)	0.086
*rs2234921(A > G)*								
Additive model								
A/A	3683	51	1.00		3222	93	1.00	
A/G	431	10	1.76 (0.87~3.55)	0.115	408	12	0.92 (0.50~28.31)	0.795
G/G	11	1	7.13 (0.94~53.81)	0.057	11	2	6.69 (1.58~28.31)	0.010
Dominant model								
A/A	3683	51	1.00		3222	93	1.00	
A/G + G/G	443	11	1.91 (0.98~3.76)	0.059	420	14	1.07 (0.60~1.89)	0.826
Recessive model								
A/A + A/G	4114	61	1.00		3630	105	1.00	
G/G	11	1	6.94 (0.92~52.40)	0.060	11	2	6.72 (1.59~28.42)	0.010

P-Y, person-years; aHR, adjusted hazard ratio; CI, confidence interval; N.A., not available. * Adjusted for age, gender, education level, cigarette smoking, and arsenic exposure.

**Table 6:** Combined effect of the *TIMP3* genotypes and arsenic exposure on the risk of skin hyperkeratosis and hyperpigmentation.

TIMP3 Genotype	Arsenic Exposure *	Keratosis/P-Y	aHR (95% CI) ^†^	*p*	Pigmentation/P-Y	aHR (95% CI)	*p*
*rs9619311*							
Dominant							
T/T	Low	8/1291	1.00		12/1300	1.00	
	High	44/2416	2.48 (1.06~5.78)	0.036	82/1945	2.89 (1.51~5.54)	0.001
T/C + C/C	Low	3/189	2.63 (0.67~10.24)	0.164	2/176	1.09 (0.24~4.93)	0.913
	High	7/229	3.81 (1.24~11.70)	0.020	11/219	2.81 (1.17~6.71)	0.021
		Trend test	1.94 (1.16~3.26)	0.012	Trend test	1.65 (1.14~2.39)	0.008
		Multiplicative test	1.53 (0.68~3.47)	0.308	Multiplicative test	0.97 (0.51~1.84)	0.924
*rs2234921*							
Dominant							
A/A	Low	8/1278	1.00		12/1280	1.00	
	High	43/2405	2.43 (1.04~5.68)	0.041	81/1941	2.82 (1.46~5.41)	0.002
A/G + G/G	Low	3/209	2.46 (0.63~9.65)	0.196	2/196	0.97 (0.21~4.41)	0.967
	High	8/234	4.30 (1.44~12.82)	0.009	12/223	2.96 (1.25~6.96)	0.013
		Trend test	2.05 (1.22~3.44)	0.007	Trend test	1.68 (1.16~2.42)	0.006
		Multiplicative test	1.76 (0.81~3.83)	0.151	Multiplicative test	1.05 (0.57~1.94)	0.878
Recessive							
A/A + A/G	Low	11/1480	1.00		14/1477	1.00	
	High	50/2634	2.02 (0.97~4.22)	0.061	91/2153	2.77 (1.51~5.06)	0.001
G/G	Low	0/0	N.A.		0/0	N.A.	
	High	1/11	14.05 (1.74~113.70)	0.013	2/11	19.82 (4.34~90.57)	<0.001
		Trend test	2.27 (1.10~4.69)	0.027	Trend test	3.12 (1.73~5.64)	<0.001
		Multiplicative test	6.95 (0.92~52.41)	0.060	Multiplicative test	7.17 (1.70~30.23)	0.007

P-Y, person-years; aHR, adjusted hazard ratio; CI, confidence interval; N.A., not available. * Low arsenic exposure indicates ≤7.7 ppm-years; ^†^ Adjusted for age, gender, education level, and cigarette smoking.

## Data Availability

Datasets of the National Cancer Registry and Death Registry System analyzed and/or generated during this study are held by the Ministry of Health and Welfare (MOHW) of Taiwan. The data are available from the corresponding author with the permission of the Taiwan MOHW.

## Supplementary Materials

The supporting information can be downloaded at https://www.mdpi.com/article/10.3390/ijms232314980/s1.
