# Supplementary material for: TIMP3 Gene Polymorphisms of -1296 T > C and -915 A > G Increase the Susceptibility to Arsenic-Induced Skin Cancer: A Cohort Study and In Silico Analysis of Mutation Impacts
Source: Int J Mol Sci. 2022 Nov 29;23(23):14980. doi: 10.3390/ijms232314980 (PMC9735753; doi:10.3390/ijms232314980)
Supplement: Supplementary file 1 [file ijms-23-14980-s001.zip › ijms-2008292-supplementary.pdf]

Table S1. Position, variants, and minor allele frequency of *TIMP3* single-nucleotide polymorphisms (SNPs) under study.

Chromosome	Position	dbSNP No.	SNP region and variants	Minor allele frequency
22	32800707	rs9619311	– 1296 T＞C	0.072
	32801088	rs2234921	– 915 A＞G	0.074
	32801104	rs2234920	– 899 T＞C	0.001

Table S2. Hazard ratios (HRs) of skin cancer in relation to diplotype of the *TIMP3* rs9619311 (T>C) and rs2234921 (A>G).

**TIMP3 Genotype**	**P-Y**	**SC Cases**	**aHR (95% CI)** *	***p***
Additive model				
T-A/T-A	13633	33	1.00	
T-A/C-G	1912	7	1.36 (0.59- 3.12)	0.474
C-G/C-G	47	0	(N.A.)	
Dominant model				
T-A/T-A	13633	33	1.00	
T-A/C-G + C-G/C-G	1959	7	1.33 (0.58- 3.05)	0.504
Recessive model				
T-A/T-A + T-A/C-G	15545	40	1.00	
C-G/C-G	47	0	(N.A.)	

P-Y, person-years; SC, skin cancer; aHR, adjusted hazard ratio; CI, confidence interval; N.A., not available. * Adjusted for age, gender, education level, cigarette smoking, and arsenic exposure.

(a) (b)

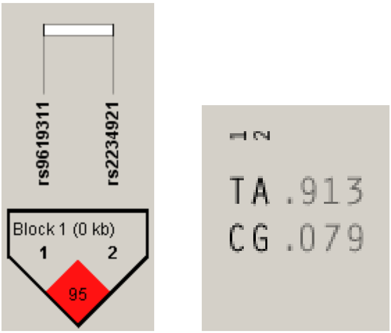

Figure S1. (a) Linkage disequilibrium and **(b)** haplotype block estimation with frequency of the two *TIMP3* polymorphisms used in the analysis.

(a)

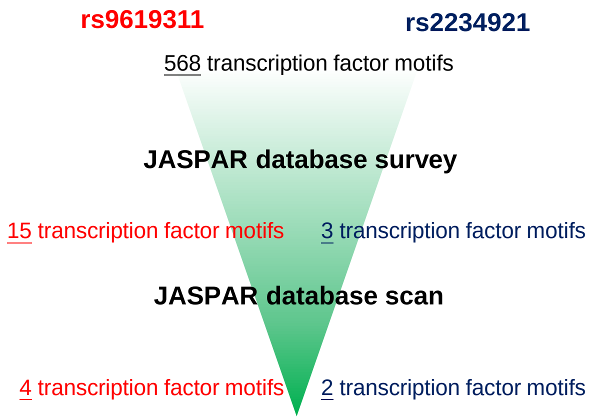

(b)

SNP site	Name	Matrix ID	Score (Wild-Type / Mutant)	Predicted sequence	Gene function
-1296 T>C (rs9619311)	BACH2	MA1101.1	5.6097364 / 0.16443755	gctgacagagctcc	Marker of DNA damage and ageing (Uittenboogaard, et al., 2013)
	MEIS2	MA0774.1	10.959028 / 8.975588	ctgacagg	Regulating cell proliferation and differentiation (Schulte D, et al., 2019)
	NFE2L2	MA0150.1	7.5447383 / 5.0879297	ctgacagggct	Regulator of defense against stress (Huppke P, et al., 2017)
	PBX2	MA1113.1	9.381758 / 8.762804	atggctgacaga	Regulating cell proliferation and differentiation (Liu Y, et al., 2021)
	NFKB	MA0105.3	4.443838 / 4.443838	agtggttccct	
	KLF14	MA0740.1	7.9426937 / 7.9426937	tgggcaggg	
	KLF16	MA0741.1	4.451736 / 4.451736	tccctgcccat	
	KLF5	MA0599.1	12.101032 / 12.101032	gctccaccct	
	KLF12	MA0742.2	7.6372814 / 7.6372814	tgggcaggg	
	KLF1	MA0493.2	10.143136 / 10.143136	agggtggag	
	MZF1	MA0056.1	6.4690514 / 6.4690514	tggggg	
	SP2	MA0516.1	9.386253 / 9.386253	gctccacccttcagc	
	SP3	MA0746.1	6.5763535 / 6.5763535	tccctgcccat	
	SP4	MA0685.2	7.330421 / 7.330421	tgggcaggg	
	YY2	MA0748.2	7.0991354 / 7.0991354	ccatggctgac	
-915 A>G (rs2234921)	E2F8	MA0865.2	-1.7847267 / -1.9462293	ctcccccagagc	Transcriptional repressor that antagonizes E2F1 (Wasserman D, et al., 2020)
	RUNX1	MA0002.1	5.7042184 / 4.366889	ttctgtgctct	Regulator of developmental hematopoiesis (Owens DDG, et al., 2022)
	TCF7L1	MA1421.1	6.9024415 / 6.9024415	ccagagcaaacg	

Figure S2. Flowchart of profiling transcription factor (TF) motifs. **(a)** A total of 568 TF motifs were surveyed followed by a comparison of TF binding sites scores between wild-type and mutant form of studied single-nucleotide polymorphisms (SNPs) using a JASPAR scan tool. **(b)** rs9619311 and rs2234921 were found to respectively be located within the predicted sequence of 15 and three TF motifs.

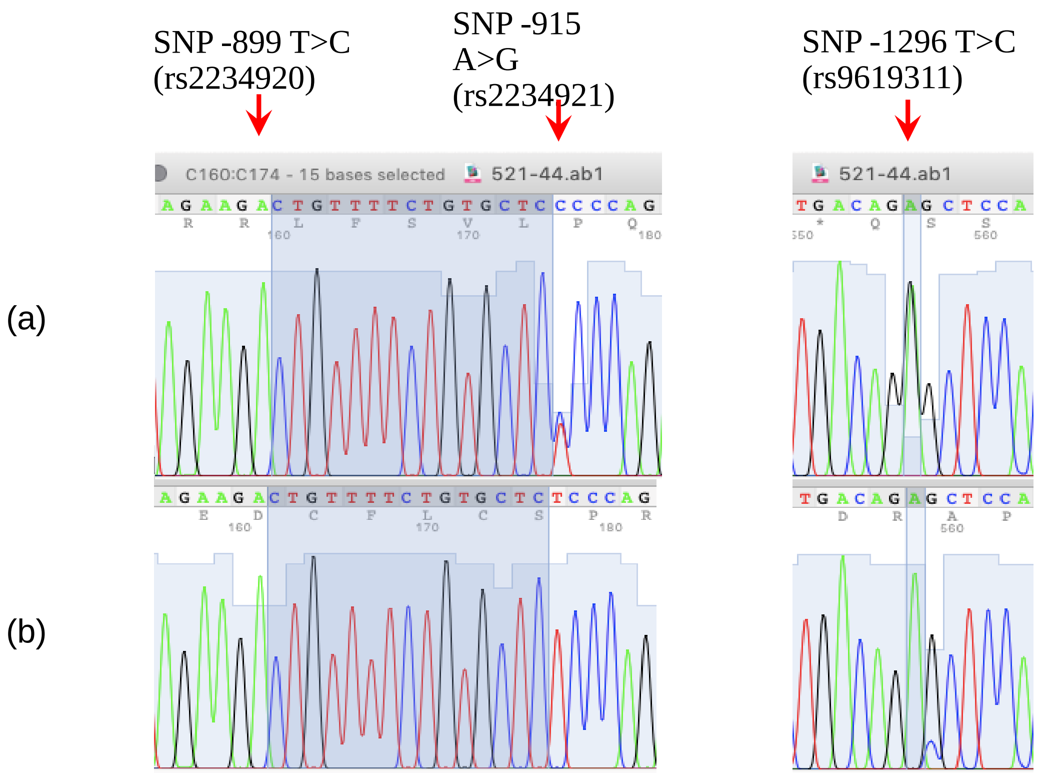

Figure S3. Representative sequencing results. (a) Individuals with genotypes TT (rs2234920), AG (rs2234921), and TC (rs9619311); (b) Individuals with genotypes TT (rs2234920), AA (rs2234921), and TT (rs9619311).
